# Prevalence of Malaria and Associated Factors among Malaria-Suspected Patients Attending Hamusit Health Center, Northwest Ethiopia: A Cross-Sectional Study

**DOI:** 10.1155/2022/1306049

**Published:** 2022-03-22

**Authors:** Getu Abeje Negatu, Getaneh Alemu Abebe, Woynshet Gelaye Yalew

**Affiliations:** ^1^Department of Biomedical Science, Samara University, Samara, Ethiopia; ^2^Department of Medical Laboratory Science, Bahir Dar University, Bahir Dar, Ethiopia

## Abstract

**Background:**

Malaria is one of the major public health problems in developing countries like Ethiopia. Despite efforts to reduce the mortality and morbidity, the disease is still a prominent health problem in Ethiopia. This study, therefore, was undertaken to assess the prevalence of malaria and associated factors among symptomatic patients in Northwest Ethiopia.

**Methods:**

A facility based cross-sectional study was conducted from February to March 2020 among 210 febrile patients attending Hamusit Health Center, Northwest Ethiopia. A structured questionnaire was used to collect data on sociodemographic characteristics and factors perceived to be associated with *Plasmodium* infection. Questionnaire data was collected through face to face interview. Thin and thick blood films were prepared from capillary blood buffy coat samples. Data were analyzed using Statistical Package for Social Sciences Software version 20.

**Results:**

Out of 210 malaria-suspected participants, 61 (29.0%) were confirmed to be infected by *Plasmodium* species. *Plasmodium falciparum* and *P. vivax* monoinfections were detected in 41 (19.5%) and 10 (4.8%) participants, respectively. Mixed infection was detected in 10 (4.8%) participants. Female participants (AOR = 2.261; 95% CI: 1.118-4.571; *P* = 0.023) and those having family members with history of malaria (AOR =2.261; 95% CI: 1.264-5.340; *P* = 0.009) had higher odds of acquiring *Plasmodium* infection as compared to their counterparts. Using insecticide-treated bed net and draining stagnant water were the most commonly mentioned malaria prevention measures reported by 71.9% and 8.1% of the respondents, respectively.

**Conclusion:**

Malaria contributes significantly for febrile illnesses in the study area. Therefore, community mobilization should be strengthen in order to improve implementation of malaria control activities and, hence, reducing the prevalence.

## 1. Background

Malaria is caused by a protozoan parasite belonging to the genus *Plasmodium. Plasmodium* (*P*) *falciparum*, *P. vivax*, *P. ovale*, *P. malariae*, and *P. knowlesi* are known to infect humans [1]. However, *P. falciparum* and *P. vivax* are the most prevalent species in the world. *P. falciparum* is the most virulent species in terms of morbidity and mortality [2, 3]. Malaria is a global public health problem; however, majority of the cases and deaths occur in the tropics and subtropics [4]. Most of the malaria cases occur in African region (90%) followed by the Southeast Asia (7%) and Eastern Mediterranean (2%) regions [5, 6]. There were an estimated 241 million malaria cases and 627,000 global malaria deaths in 2020 compared to 227 million cases in 2019—an increase of about 14 million cases and 69,000 deaths over the previous year. About 95% of all malaria cases and deaths were in the World Health Organization (WHO) African Region [7]. In Ethiopia, 68% of the population lives in malaria risk areas [8].

Humans are primarily infected through the bite of an infected female *Anopheles* mosquito that inoculates sporozoites during a blood meal [9]. After inoculation, parasites circulate with blood and those reaching to the liver undergo one cycle development. Then, parasites infect and multiply inside red blood cells to bring the characteristic sign and symptoms including fever, headache, and vomiting which usually appears between 10 and 15 days after the mosquito bite [10]. If not treated, malaria might become life-threatening by disrupting blood supply to vital organs [11]. Complications responsible for severe malaria include occurrence of anemia, impaired consciousness, respiratory distress, hypoglycemia, and jaundice [12].

The Ethiopian Federal Ministry of Health has been making efforts to reduce malaria morbidity and mortality through utilization of insecticide-treated nets (ITNs), environmental control, chemoprophylaxis, and rapid case detection followed by prompt case management [13]. Hence, the disease incidence shows a decline in the country. However, the parasite incidence has been increasing in certain focal areas within the last years. Therefore, periodic assessment of malaria prevalence in endemic areas helps to evaluate the existing interventions. Hamusit, located in Northwest Ethiopia, is among the top five malaria-affected districts in Amhara Region. However, an updated data on the malaria prevalence and associated factors is lacking in the area. Therefore, this study was undertaken to assess the prevalence of malaria and associated factors among malaria-suspected patients attending Hamusit Health Center.

## 2. Methods and Materials

### 2.1. Study Design and Setting

A facility-based cross-sectional study was conducted at Hamusit Health Center, Northwest Ethiopia, from February to March 2020. Hamusit town is administratively part of Dera district, and it is located at geographic coordinates of 11° 43′ 0^″^N and 37° 38′ 0^″^E. The climate of the area is woyna dega and has an average annual rain fall of 1300 mm and mean annual temperature of 26°C (source: *Woreda* office of agriculture). The health center serves for 55,422 people in the catchment area. Owing to low altitude and irrigation activities, malaria is a common public health problem in the area.

### 2.2. Study Population

The sample size was calculated using single population proportion formula based on the 95% confidence limits, 4% margin of error, malaria prevalence of 8.7% from previous study conducted in Northeast Ethiopia [14], and 10% nonresponse rate. Accordingly, the calculated sample size was 210. Patients who visited the health center laboratory for blood film examination and met the eligibility criteria were recruited by systematic random sampling technique considering the case flow in the health center from February to March of the previous year. Febrile patients clinically suspected of malaria who were 5 years old or above and willing to participate in the study were included. Patients who took antimalarial and antibiotics therapy with in the past one month before enrollment and those who were unable to respond to research questions were excluded from the study.

### 2.3. Data Collection

A structured and pretested questionnaire was adapted from the “National Malaria Indicator Survey Household Questionnaire” [15] to be used in the present study. It was administered to gather information on sociodemographic characteristics and clinical data of study participants. Data on malaria control activities including indoor residual spraying (IRS) in the past 12 months, number of ITNs owned per house hold, presence of hole in the wall, presence of surface water with in 500 m from home, type of surface water, distance of surface water from home, and habit of sleeping or working outside at night were also collected.

Capillary blood was obtained via finger puncture. Each patient's finger was cleaned with 70% ethyl alcohol, and the side of preferably ring fingertip was pricked with a sterile lancet. After wiping away the first drop, capillary blood was collected by filling three-fourth length of a heparinized capillary tube. The capillary tube containing blood was centrifuged at 1200 revolution per minute for 5 minutes, and the buffy coat and the red cell layer just below the plasma (approximately 1-2 mm) were aspirated using a micropipette for thin and thick blood film preparation. Buffy coat samples were examined in order to increase the sensitivity of conventional blood film microscopy, because infected red blood cells tend to concentrate in and just below the buffy coat. We have shown in our recent publication that capillary blood buffy coat smear has significantly superior detection rate [16].

Thin and thick blood films were simultaneously prepared from each participant and stained with 10% Giemsa for 10 minutes and then examined microscopically following standard protocol explained elsewhere [17]. Asexual parasite stages and gametocytes were counted separately from each positive smear against 500 and 1000 WBCs, respectively.

In order to ensure data quality, standard operating procedures were strictly followed and quality of Giemsa staining reagent was checked every week using known positive and negative blood samples obtained from Amhara Public Health Institute (regional reference laboratory). Moreover, slides were read by two laboratory personnel where each reader was blind to results of the other. Both malaria microscopists are certified for “malaria microscopy and quality assurance from the National Medical Parasitology Reference Laboratory” and had more than two years of practical experience at health institutions in malaria endemic areas. Discrepant results were examined by malaria laboratory experts from Amhara Public Health Institute.

### 2.4. Statistical Analysis

Data were coded, entered, cleaned, and analyzed using Statistical Package for Social Science Software version 20. Descriptive statistics like frequency, percentage, and mean were manipulated to explain the study participants and to show the malaria prevalence in the study area. Binary logistic regression was run to assess association between sociodemographic and other independent variables with *Plasmodium* infection. Odds ratio (OR) with the corresponding 95% confidence interval (CI) was used to determine the strength in association between dependent and independent variables. Associations were considered as significant only if *P* value was less than 0.05.

### 2.5. Ethical Considerations

Ethical approval was obtained from Bahir Dar University, College of Medicine and Health Sciences Ethical Review Committee with project code of 008/2020 prior to the commencement of the study. Permission letter was obtained from Amhara Public Health Institute, and support letter was also obtained from South Gonder Zonal Department, Dera Woreda Health Office, and Hamusit Health Center. Informed written consent was obtained from participants with age ≥ 18 years old. For participants of age < 18 years old, informed written consent was obtained from their parents or guardians. Study participants with positive results for any hemoparasite were linked to the health center for appropriate treatment.

## 3. Results

Among 210 study participants, 105 (50%) were males while the rest 105 (50%) were females. Regarding to their age composition, 153 (72.9%) and 57 (27.1%) participants were >15 and 6-15 years old, respectively. One hundred ninety-three (91.1%) participants were rural residents while the remaining 17 (8.1%) were urban dwellers. One hundred and eighteen (56.2%) participants had family size ≥ 5 (Table 1).

One hundred seventy-seven (84.3%) participants had axillary temperature of 37.5°C or above. Ninety-six (45.7%) and 114 (54.3%) had malaria history and had family members with history of malaria with in the previous 1 year, respectively. Two hundred three (96.7%) participants had fever while 179 (85.2%) and 191 (91%) patients complained for headache and nausea, respectively (Table 2).

Insecticide-treated bed net and draining stagnant water were the most commonly mentioned malaria prevention measures reported by 71.9% and 8.1% of the respondents, respectively. Forty-one (27.1%) patients responded that their households owned two ITNs. Nearly one-third (31.5%) of the participants responded that their homes were sprayed with IRS within a year before the present data collection period (Table 3).

Overall, 61 (29.0%) participants were confirmed to be infected by *Plasmodium* species. *Plasmodium* distribution at species level revealed that *P. falciparum* monoinfection accounted for the highest frequency, detected in 41 (19.5%) participants. Asexual parasite stages were detected in 59 participants while only gametocytes were detected in 2 patients (Table 4).

The prevalence of malaria was 21.9% in males and 36.2% in females. Females were 2.261 times more likely to be infected with malaria than males (AOR = 2.261; 95%CI = 1.118‐4.571). In addition, individuals who had family members with history of malaria were about 2.598 times more likely to be infected with malaria than those having family members with no history of malaria (AOR = 2.598; 95%CI = 1.26‐5.340). The distribution of malaria was higher among rural residents (29.5%) than among urban residents (23.5%); however, the difference was not statistically significant (Table 5).

## 4. Discussion

Malaria is a major public health problem widespread throughout tropical and subtropical regions of the world including Ethiopia. In the present study, the overall prevalence of malaria infection (29.0%) was lower than previous study findings of 33.8% to 53.68% in Ethiopia [18–20]. The present results were also lower than prevalence of 64.5%, 32.4%, and 41.6% from Tanzania [21], Liza [22], and Nigeria [23], respectively. The low prevalence in the present study might be due to difference in the study period. Data for the present study was collected during the minor malaria transmission season. Variation in implementation of malaria intervention activities and difference in the local epidemiology of malaria parasites might also be responsible. However, the present prevalence was higher than similar study results from different geographical settings of Ethiopia where the prevalence ranged from 4.7% to 25.8% [24–29]. It was also higher than 27.6% prevalence reported from India [30]. Malaria parasitemia in the present study was also higher as compared to similar study results from Bangladesh (0.76%, 3/400) [31], India (0.08%, 14/17209) [30], and North Ethiopia (1%, 6/600) [27]. The higher prevalence in the present study might be due to application of buffy coat examination in the present study, which is more sensitive technique than conventional blood film examinations [16]. Moreover, variations in the national or local transmission of the disease across different geographical settings also matter.

Majority of the infections (19.5%, 41/210) in the present study were caused by *P. falciparum* while each of *P. vivax* and mixed infections contributed for 4.8% (10/210) of the infections. This was in agreement with previous study finding from Arsi Negele, Southern Ethiopia, where the prevalence of *P. falciparum*, *P. vivax*, and mixed infections were 19.8%, 7.4%, and 6.2%, respectively [32]. The species level distribution in the present study is in line with the national distribution in Ethiopia where *P. falciparum* accounts more than 60% of the infections while *P. vivax* is responsible for approximately 40% of the infections [13, 14].

According to the present study, individuals who had family history of malaria within one year were 2.598 times more likely to be infected by *Plasmodium* species as compared to their counterparts (*P* = 0.009). This might be due to the fact that family members with history of malaria infection may become reservoirs for *Plasmodium* parasites and serve as sources of infection for the rest of the family members. In addition, the present data showed that females were 2.261 times more likely to get infected as compared to males (*P* = 0.023). On the contrary, a study conducted at Kola Diba Health Center reported that males were more affected than females [18]. Variations in nature of occupation and outdoor activities among males and females might bring such differences. All the other analyzed factors (Table 5) were not significantly associated with *Plasmodium* parasitemia. However, it is difficult to justify why those factors were not associated because we have recruited small number of participants. Moreover, we collected data at health institution which was not supplemented with observation to increase the data quality like how they are using ITN. The present study has certain limitations that we recruited small sample size, data was collected in the minor malaria transmission season, and the study was institution based that it does not indicate the actual prevalence in the community.

## 5. Conclusions

The prevalence of malaria parasitemia among febrile patients attending Hamusit Health Center is considerable. Moreover, *P. falciparum* monoinfection was responsible for the majority of the infections in the area. Individuals with female sex and those having family history of malaria infection were at higher risk of *Plasmodium* infection. Therefore, efforts must be made to expand and sustain the combined application of ITNs, IRS, and larval source management at Hamusit town and the rural localities. In addition, encouraging family members infected with *plasmodium* species to seek timely treatment is also important to minimize the risk of infection among other family members.

## Figures and Tables

**Table 1:** Sociodemographic characteristics of (*N* = 210) of study participants attending Hamusit Health Center, Northwest Ethiopia, from February to March 2020.

Variable	Category	Frequency	Proportion (%)
Age group (in years)	6-15	57	27.1
	>15	153	72.9

Sex	Male	105	50
	Female	105	50

Residence	Urban	17	8.1
	Rural	193	91.9

Family size	<5	92	43.8
	≥5	118	56.2

Occupation	Farmer	148	70.5
	Student	51	24.3
	Private business	6	2.9
	Employed	5	2.4

**Table 2:** Clinical data of study participants among febrile patients attending Hamusit Health Center, Northwest Ethiopia, from February to March 2020.

Variable	Category	Frequency	Proportion (%)
Malaria history within the previous 1 year	Yes	96	45.7
	No	114	54.3

Family members with history of malaria	Yes	114	54.3
	No	96	45.7

Axillary temperature (°C)	>37.5	177	84.3
	≤37.5	33	15.7

Duration of the present fever/illness	≤24 hrs	147	70.0
	>24 hrs	63	30.0

Participants with fever	Yes	203	96.7
	No	7	3.3

Sweating	Yes	179	85.2
	No	31	14.8

Headache	Yes	191	91.0
	No	19	9.0

Nausea	Yes	160	76.2
	No	50	23.8

Vomiting	Yes	60	28.6
	No	150	71.4

Weakness	Yes	179	85.2
	No	31	14.8

Loss of appetite	Yes	163	77.6
	No	47	22.4

**Table 3:** Malaria control activities and conditions among febrile patients attending Hamusit Health Center, Northwest Ethiopia, 2020.

Variable	Category	Frequency	Proportion (%)
Availability of ITN	Yes	151	71.9
	No	59	28.1

Reason for unavailability of ITN	Never received	26	44.1
	Lost/given to somebody else	10	16.9
	Old and thrown away	23	39.0

Proportion of one ITN to family members	For 1-1.5 persons	41	27.1
	For 1.6–2.5 persons	64	42.4
	For more than 2.5 persons	46	30.5

Habit of sleeping under ITN	Always	127	60.5
	Sometimes	24	11.4
	Never	59	28.1

House sprayed with IRS within 12 months	Yes	65	31.0
	No	145	69.0

Time of IRS spray	<6 months ago	21	32.3
	≥6 months ago	44	67.7

Presence of hole in the wall	Yes	92	43.8
	No	118	56.2

Presence of surface water with in 500 m from home	Yes	160	76.2
	No	50	23.8

Type of surface water	River	140	66.7
	Lake/pond	3	1.4
	Swamp/small stagnant water	17	8.1

Distance of surface water from home (meter)	0-100	125	78.1
	101-500	35	21.9
Habit of sleeping or working outside at night	Yes	54	25.7
	No	156	74.3

**Table 4:** Prevalence of malaria parasites among febrile patients attending Hamusit Health Center, Northwest Ethiopia, from February to March 2020.

		Total examined	Number positive	Prevalence (in %)
Cumulative	Overall prevalence		61	29.0
	*P. falciparum* only	210	41	19.5
	*P. vivax* only		10	4.8
	Mixed infection		10	4.8

Parasite stage detected	Trophozoite only		44	72.1
	Schizont only	61	1	1.6
	Gametocyte only		2	3.3
	More than 1 stage		14	23.0

^∗^Mixed infection; presence of both P. falciparum and P. vivax at the same time from a single participant.

**Table 5:** Bivariate and multivariate logistic regression analysis of factors associated with malaria among febrile patients attending Hamusit Health Center, Northwest Ethiopia, from February to March 2020.

Variables	Category	Number examined	Rate of Plasmodium infection *N* (%)	COR (95% CI)	*P* value	AOR (95% CI)	*P* value
Sex	Male	105	23 (21.9)	1			
	Female	105	38 (36.2)	2.022 (1.099-3.722)	0.024	2.261 (1.118-4.571)	*0.023*

Age group (years)	6-15	57	32 (56.1)	5.473 (2.825-10.602)	*P* < 0.001	2.463 (0.723-8.394)	0.150
	>15	153	29 (19.0)	1			

Residence	Urban	17	4 (23.5)	1			
	Rural	193	57 (29.5)	1.362 (0.426-4.356)	0.602		

Family size	<5	92	26 (28.3)	1			
	≥5	118	35 (29.7)	1.070 (0.586-1.954)	0.825		

Malaria history within the previous 1 year	Yes	96	28 (29.2)	1.011 (0.556-1.838)	0.972		
	No	114	33 (28.9)	1			

Family members with history of malaria	Yes	114	44 (38.6)	2.921 (1.532-5.571)	0.001	2.598 (1.264-5.340)	*0.009*
	No	96	17 (17.7)	1			

Habit of sleeping under ITN	Always	127	41(32.3)	1			
	Sometimes	24	7 (29.2)	0.816 (0.316-2.107)	0.674	1.134 (0.378-3.401)	0.822
	Never	59	13 (22.0)	0.620 (0.301-1.276)	0.194	0.947 (0.419-2.140)	0.895

House sprayed with IRS within 12 months	Yes	65	15 (23.1)	1			
	No	145	46 (27.6)	1.549 (0.789-3.041)	0.204	1.395 (0.642-3.032)	0.400

Presence of hole in the wall	Yes	92	28 (30.4)	1.127 (0.619-2.051)	0.696		
	No	118	33 (28.0)	1			

Presence of surface water within 500 m from home	Yes	160	53 (33.1)	2.600 (1.140-5.932)	0.023	2.225 (0.884-5.597)	0.809
	No	50	8 (16.0)	1			

Habit of sleeping or working outside at night	Yes	54	16 (29.6)	1.039 (0.527-2.048)	0.913		
	No	156	45 (28.8)	1			

AOR: adjusted odd ratio; CI: confidence interval; COR: crude odd ratio.

## Data Availability

The original data for this study is available from the corresponding author.

## Abbreviations

FMOH: Federal Ministry of Health
HHs: Households
IRS: Indoor residual spraying
ITNs: Insecticide-treated mosquito nets
PCR: Polymerase chain reaction
WHO: World Health Organization.

## References

[1] WHO (2012). World malaria report 2011. https://www.mmv.org/newsroom/publications/world-malaria-report-2011?gclid.

[2] Ferri (2009). Protozoal infections. *Ferri's Color Atlas and Text of Clinical Medicine*.

[3] WHO (2015). Guidelines for the treatment of malaria. https://apps.who.int/iris/handle/10665/162441.

[4] WHO (2017). World malaria report 2017. https://www.mmv.org/newsroom/publications/world-malaria-report-2017?gclid.

[5] Alonso P., Noor A. M. (2017). The global fight against malaria is at crossroads. *The Lancet*.

[6] WHO (2017). World malaria report. https://www.who.int/malaria/publications/world-malaria-report-2017.

[7] WHO (2021). *World Malaria Report*.

[8] Federal Ministry of Health (FMOH) (2006). *National five-year strategic plan for malaria prevention and control in Ethiopia 2006-2010*.

[9] Kayser (2005). *Medical Microbiology, part V(Parasitology)*.

[10] Antia M., Herricks T., Rathod K. (2007). Microfluidic modeling of cell−cell interactions in malaria pathogenesis. *PLoS Pathogens*.

[11] Clark I., Cowden W. (2003). The pathophysiology of *Plasmodium falciparum* malaria. *Pharmacology and Therapeutics*.

[12] Schellenberg D., Menendez C., Kahigwa E. (1999). African children with malaria in an area of intense *Plasmodium falciparum* transmission: features on admission to the hospital and risk factors for death. *American Journal Tropical Mediccine Hygine*.

[13] Jones G., Steketee R. W., Black R. E., Bhutta Z. A., Morris S. S. (2003). How many child deaths can we prevent this year. *The Lancet*.

[14] Biniyam B. (2019). *A Four Year Trend Analysis of Malaria Prevalence in Aysaita Primary Hospital, Aysaita Woreda, Afar Regional State, Northeast Ethiopia, [Ph.D thesis]*.

[15] Ethiopian Public Health Institute (2016). Ethiopia national malaria indicator survey 2015. https://www.malariasurveys.org/documents/Ethiopia_MIS_2015.pdf.

[16] Abeje G., Gelaye W., Alemu G. (2021). Comparison of capillary, venous and buffy coat blood samples in detecting *Plasmodium* species among malaria suspected patients attending at Hamusite health center. a cross-sectional study. *BMC Infectious Diseases*.

[17] Bisoffi Z., Gobbi F., Buonfrate D., Van den Ende J. (2012). Diagnosis of malaria infection with or without disease. *Mediterr Journal of Hematolology Infectious Disease*.

[18] Alemu A., Muluye M., Mihret M., Adugna M., Gebeyaw M. (2012). Ten year trend analysis of malaria prevalence in Kola Diba, North Gonder North west Ethiopia. *Parasites and Vectors*.

[19] Haque U., Ahmed S. M., Hossain S. (2009). Malaria prevalence in endemic districts of Bangladesh. *PloS One*.

[20] Tesfaye (2017). Prevalence of malaria among patients attending.

[21] Yimer F., Animut A., Erko B., Mamo H. (2015). Past five-year trend, current prevalence and household knowledge, attitude and practice of malaria in Abeshge, south-central Ethiopia. *Malaria Jornal*.

[22] Sumari D., Mwingira F., Selemani M., Mugasa J., Mugittu K., Gwakisa P. (2017). Malaria prevalence in asymptomatic and symptomatic children in Kiwangwa, Bagamoyo district, Tanzania. *Malaria Journal*.

[23] Huang F., Takala-Harrison S., Liu H. (2017). Prevalence of clinical and subclinical *Plasmodium falciparum* and *Plasmodium* vivax malaria in two remote rural communities on the Myanmar–China border. *American Journal of Tropical Medicine Hygine*.

[24] Fana S. A., Anka S. A., Imam A. U., Nataala S. U. (2015). Prevalence and risk factors associated with malaria infection among pregnant women in a semi-urban community of north-western Nigeria. *Infectious Disease of Poverty*.

[25] Tadesse F., Fogarty A. W., Deressa W. (2018). Prevalence and associated risk factors of malaria among adults in East Shewa Zone of Oromia Regional State, Ethiopia: a cross-sectional study. *BMC Public Health*.

[26] Delil R. K., Dileba T. K., Habtu Y. A., Gone T. F., Leta T. J. (2016). Magnitude of malaria and factors among febrile cases in low transmission areas of Hadiya zone, Ethiopia: a facility based cross sectional study. *PLoS One*.

[27] Molla E., Ayele B. (2015). Prevalence of malaria and associated factors in Dilla town and the surrounding rural areas, Gedeo Zone, Southern Ethiopia. *Journal of Bacteriology & Parasitology*.

[28] Debo G. W., Kassa D. H. (2016). Prevalence of malaria and associated factors in Benna Tsemay district of pastoralist community, Southern Ethiopia. *Tropical Diseases, Travel Medicine and Vaccines*.

[29] Gebretsadik D., Feleke D. G., Fiseha M. (2018). Eight-year trend analysis of malaria prevalence in Kombolcha, South Wollo, north-central Ethiopia: a retrospective study. *Parasites Vectors*.

[30] Abebaw A. (2019). *The prevalence of symptomatic and asymptomatic malaria and its associated factors in Debre Elias district communities, northwest Ethiopia, [Ph.D thesis]*.

[31] Chourasia M. K., Raghavendra K., Bhatt R. M., Swain D. K., Valecha N., Kleinschmidt I. (2017). Burden of asymptomatic malaria among a tribal population in a forested village of central India: a hidden challenge for malaria control in India. *Public Health*.

[32] Mengistu H. (2014). Trend analysis of malaria prevalence in Arsi Negelle Health Center Southern Ethiopia. *Acadamic Journal*.

